# Octreotide attenuates intestinal ischemia/reperfusion mischief in rats through modulation of Nrf2/PRX2/ASK1/JNK signaling pathway

**DOI:** 10.1007/s00210-025-04157-0

**Published:** 2025-05-01

**Authors:** Nermein F. El Sayed, Diaa Ragab, Walied Abdo, Mai El-Sayed Ghoneim

**Affiliations:** 1 Department of Pharmacology and Toxicology, Faculty of Pharmacy, Ahram Canadian University, Giza, Egypt; 2 Department of Pharmacology and Toxicology, Faculty of Pharmacy, University of Sadat City (USC), Sadat City, 32897 Egypt; 3 Department of Pathology, Faculty of Veterinary Medicine, Kafrelsheikh University, Kafr El-Sheikh, 33516 Egypt; 4 Department of Pharmacology and Toxicology, Faculty of Pharmacy, University of Sadat City (USC), Sadat City, 32897 Egypt

**Keywords:** Octreotide, IIR, Antioxidant, Autophagy, Apoptosis, Inflammation

## Abstract

Intestinal ischemia/reperfusion (IIR) is a substantial cause of mortality and morbidity worldwide. Octreotide (OCT) has been proven to be effective against various organ insults. However, the exact mechanism by which it exerts protective effect against IIR is still obscure. Thus, the aim was to unveil the potential role of octreotide in an IIR model and decipher its mechanism of action. The rats were allocated into sham-operated, IIR, and OCT groups. Histopathological changes were performed to assess the intestinal injury. Immunohistochemical analysis was used to estimate the NF-κB, Bcl2, caspase-3, IL-17, LC3B, and beclin-1. The mRNA of TNF-α and IL-17 were examined using real time PCR. The levels of p-Nrf2, PRX2, p-JNK, ASK1, and LC3 were assessed using western blot technique. The levels of total antioxidant capacity and SOD were measured using appropriate kits. Furthermore, the protein expressions of Bax, caspase-3, ASK1, and Nrf2 were assessed using proper ELISA kits. Additionally, the comet assay was determined to investigate the effect on apoptosis. At the molecular level, OCT administration upregulated TAC and SOD levels, demonstrating its antioxidant effect. The anti-apoptotic effect was signified by the upregulation of Bcl2 and downregulation of Bax and caspase-3, which was confirmed by comet assay. Furthermore, OCT decreased the levels of TNF-α, NF-κB, and IL-17, confirming its anti-inflammatory effect. OCT pre-treatment triggered autophagy, as evidenced by the upregulation of beclin-1 and LC3B. These effects were accomplished by increasing p-Nrf2 and PRX2 and decreasing ASK1 and p-JNK. Consequently, this impeded the necrosis of intestinal cells and improved the intestinal histoarchitecture abnormalities. Ultimately, OCT successfully ameliorated IIR injury via modulating the Nrf2/PRX2/ASK1/JNK signaling trajectory, leading to autophagic, antioxidant, anti-apoptotic, and anti-inflammatory effects.

**Graphical abstract:**

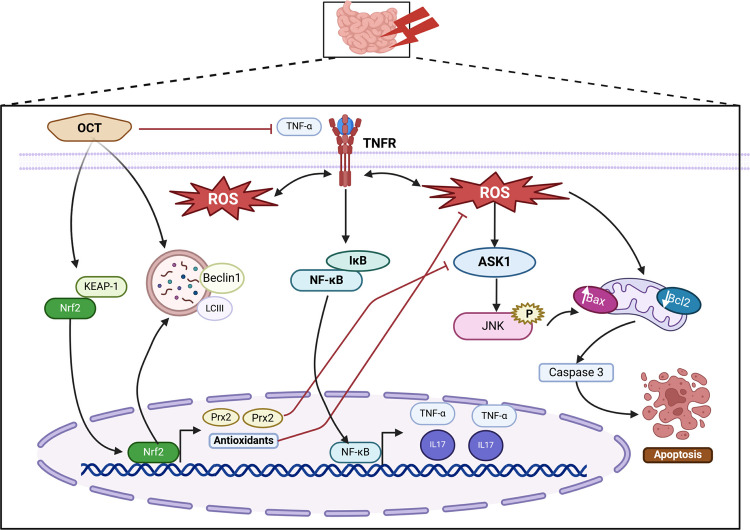

**Supplementary Information:**

The online version contains supplementary material available at 10.1007/s00210-025-04157-0.

## Introduction

Intestinal ischemia/reperfusion (IIR) is one of the leading life-threatening conditions worldwide that leads to a systemic inflammatory response and multiple organ dysfunction syndromes (Yasuhara 2005). Intestinal reperfusion aggravates damage in many clinical situations, such as intestinal obstruction, strangulation, mesenteric artery thrombosis, trauma, shock, and intestinal transplantation (Mallick et al. 2004). The mechanisms involved in IIR injury are complex, as reactive oxygen species (ROS) are generated followed by an inflammatory response upon reperfusion, leading to destruction of the intestinal mucosal barrier, increased vascular permeability, bacterial translocation, and the release of inflammatory mediators and apoptotic factors (Chen et al. 2020). Hence, novel drugs must be developed to safeguard against these detrimental impacts.

Nuclear factor erythroid 2-related factor 2 (Nrf2) plays a pre-emptive role in several diseases, including IR injury, by coordinating the controlled expression of antioxidant genes to restore redox homeostasis (Bellezza et al. 2018). It also induces the expression of peroxiredoxin 2 (PRX2) (Kensler et al. 2007), a member of the ROS family that functions by eliminating peroxides and inhibiting apoptosis (Leak et al. 2013). Under stress, depletion of PRX2 stimulates oxidative injury by increasing the level of apoptosis signal regulating kinase 1 (ASK1), which consequently triggers c-Jun N-terminal kinase (JNK), ultimately inducing apoptosis (Ichijo et al. 1997; Saitoh et al. 1998; Matsuzawa et al. 2005). Therefore, activating the Nrf2/JNK signaling pathway may protect against IIR insult.

Octreotide (OCT) is an octapeptide analog of endogenous somatostatin (Katz and Erstad 1989). OCT has beneficial impacts on IR injury in a variety of organs, including the retina (Wang et al. 2015), kidney (Xu et al. 2017), pancreas (Woeste et al. 2008), liver (Mohamed et al. 2021), and ovary (Yildirim et al. 2015). Additionally, some studies highlighted the prophylactic action of OCT following intestinal IR injury (Morris et al. 1993; Takano et al. 2012). However, the precise molecular pathways involved in the potential protective role of the PRX2 signaling pathway against IIR insults remain unclear.

Therefore, the overarching goal of this study was to elucidate the involvement of the Nrf2/PRX2/ASK1/JNK signaling trajectory and to highlight the protective role of OCT against IIR-induced insult.

## Materials and methods

### Animals

Adult male Wistar albino rats (150–200 g) were purchased from the animal center, Faculty of Pharmacy, Ahram Canadian University (Giza, Egypt). One week prior to the experiment, all rats were kept under a 12-h light/dark cycle, controlled temperature (25 ± 1 °C), and humidity (55 ± 5%), and had free access to food and tap water. All the conditions strictly followed the recommendations of the National Institutes of Health (National Research Council 2011).

### Surgical procedure

The IIR injury was performed as previously described by Jacob and his colleagues (Jacob et al. 2003). Briefly, 12-h fasting rats were anesthetized via an intraperitoneal injection (i.p) of 100 mg/kg ketamine (Troikaa Pharmaceuticals Ltd., Gujarat, India) and 10 mg/kg xylazine (Adwia Co., 10 th of Ramadan, Egypt) cocktail (Atef et al. 2017). A median laparotomy was performed, and the superior mesenteric artery (SMA) was exposed to be clamped by a nontraumatic microvascular clamp for 1 h to induce ischemia. Intestinal ischemia was detected when the color of the intestine turned pale. The clamp was then removed at the end of the ischemic period to start reperfusion for 2 h, which was validated by the restoration of pulse and color. The midline incision was then closed. The body temperature was maintained at 36.5 ± 0.5 °C with a heat lamp throughout the surgical operation.

### Experimental design

Eighteen rats were randomly allocated into three groups (*n* = 6/group). The first was assigned to the sham-operated (SO) group, in which the rats underwent clamp-free surgery. The second group was the IIR group, in which the rats were subjected to 1 h of ischemia followed by 2 h of reperfusion. The animals in these groups received saline 30 min prior to anesthesia. Finally, in the third group, the rats were injected with octreotide (50 µg/kg; i.p.; OCT group) (Shanghai TASH Biotechnology Co., Ltd., Shanghai, China) 30 min before IIR was induced (Kaçmaz et al. 2004).

### Sample preparation

At the end of the reperfusion, the rats were euthanized, and a 6-cm-long terminal ilea from the intestines were isolated and divided into four samples (1.5 cm each). The first was preserved in 10% buffered formalin for histopathological and immunohistochemical (IHC) studies. Another sample was kept in radioimmunoprecipitation assay (RIPA) buffer supplemented with a complete protease inhibitor cocktail (Bio Basic, Ontario, Canada; Cat# PL008 - 5) for western blotting measurements, while the third sample was homogenized in phosphate-buffered saline (PBS) for biochemical estimations. Finally, the last sample was kept in RNA lysis buffer for real-time quantitative polymerase chain reaction (qPCR) measurements. All specimens were aliquoted and stored at − 80 °C.

### Histopathological examination

The formalin-preserved intestinal samples were dehydrated in a series of ascending grades of ethyl alcohol, cleared in xylene, and embedded in melted paraffin wax. After blocking, 5-µm-thick sections were cut, stained with hematoxylin and eosin (H&E), and blindly examined for histopathological changes using a light microscope (Leica Microsystems microscope, adapted with a Leica DFC camera). Tissue injuries in the intestinal mucosa were evaluated according to the criteria described by Chiu et al. (1970) and graded from zero to five (Chiu et al. 1970).

### Immunohistochemical analysis

Immunohistochemical staining was performed as described by Khalil et al. (2020). Sections were dewaxed, retrieved in 0.05 M citrate buffer (pH 6.8), and treated with 0.3% H2O2. Then, the cells were incubated with polyclonal anti-caspase- 3 antibodies (Invitrogen, USA, Cat# PA5 - 77,887, dilution 1:100); anti-LC3B antibody (Abcam, USA, Cat#ab48394, dilution 1:100); rabbit polyclonal antibody against Beclin 1 (Thermo Scientific, Denmark, #PA595184, dilution 1:200); IL17 A antibody (Invitrogen, USA, PA5 - 79470, dilution 1:200); anti-NF-ĸB P65 (Santa Cruz, USA, Cat# (F- 6): sc- 8008, dilution 1:100); rabbit monoclonal anti-bcl2 (Abcam, USA, Cat# ab182858, dilution 1:500); rabbit monoclonal anti-bcl2 (Abcam, USA, Cat# ab182858, dilution 1:500). After being rinsed with phosphate-buffered saline, the sections were incubated with a goat anti-rabbit secondary antibody (Cat# K4003, EnVision + ™ System Horseradish Peroxidase Labeled Polymer, Dako) for 30 min at room temperature. The slides were then visualized with a DAB kit and stained with Mayer’s hematoxylin as a counterstaining agent. The IHC results were assessed as the percentage of positive cells per mm^2^ using ImageJ analysis software through assessment of the threshold difference, and the labeling index was calculated as the percentage of positive cells among the total 1000 cells.

### Western blot analysis

The RIPA-preserved intestine samples were homogenized, and their protein concentrations were quantified using a Bradford protein assay kit (SK3041:1; Bio Basic, Ontario, Canada). A sample containing 20 µg of protein was mixed with an equal volume of 2 × Laemmli buffer, separated by sodium dodecyl sulfate–polyacrylamide gel electrophoresis (SDS-PAGE) using the TGX Stain-Free™ FastCast™ Acrylamide Kit (Bio-Rad, CA, USA; Cat #161–0181), and then transferred to a nitrocellulose membrane. The membrane was subsequently treated with Tris-buffered saline containing Tween 20 (TBST) and bovine serum albumin (BSA 3%) at room temperature to block nonspecific antibody binding. One hour later, the blocking solution was removed, and the membrane was incubated with primary antibodies overnight at 4 °C. The primary antibodies used were against pS344-Nrf2 (ThermoFisher Scientific, USA, Cat # PA5 - 105664, dilution 1:500); PRX2 (Santa Cruz Biotechnology, USA, Cat # MA5 - 35480, dilution 1:1000); pThr845-ASK1 (Cell Signaling Technology, USA, Cat# 3765, dilution 1:1000); pT185-JNK (Cell Signaling Technology, USA, Cat# 9251, dilution 1:1000); LC3 (ThermoFisher Scientific, USA, Cat # PA1 - 16931, dilution 1:1000); β-actin (Cell Signaling Technology, USA, dilution 1:1000). The membrane was then washed four times with TBST (5 min for each) and incubated with horseradish peroxidase (HRP)–conjugated secondary antibody (Sigma-Aldrich Chemicals, St. Louis, MO, USA, dilution 1:1000) for 1 h at room temperature. The chemiluminescent substrate (ClarityTM Western ECL substrate—Bio-Rad, CA, USA; Cat #170–5060) was then added, and the resultant bands were quantified for intensity using Image Lab™ software version 5.1 (Chemi Doc™ imaging system; Bio-Rad, CA, USA).

### Total antioxidant capacity and superoxide dismutase activity assessments

Total antioxidant capacity (TAC) was assessed as described by Benzie and Strain (1996). Its assessment is based on measuring the absorbance (593 nm) of the blue-colored complex that results from the reduction of the ferric tripyridyltriazine (Fe3 + -TPTZ) by the homogenate antioxidants at pH = 3.6. Superoxide dismutase (SOD) intestinal activity was assayed using BioVision kit (Massachusetts, US; Cat # K335 - 100). Briefly, the PBS homogenate was centrifuged at 14,000 × g for 5 min at 4 °C, after which the cell debris was discarded. Then, 20 µl of the supernatant was mixed with water-soluble tetrazolium salts (WSTS) and a superoxide anion that cleaved the WSTS into formazan dye. The SOD activity in the sample was inversely proportional to the formazan dye absorbance, which was measured spectrophotometrically at 450 nm.

### Real-time quantitative PCR (qPCR)

Intestinal tumor necrosis factor alpha (TNF-α) gene expression was assessed using qPCR analysis. Total RNA was extracted from homogenized tissues from all the different groups using Direct-zol RNA Miniprep Plus (Zymo Research Co., Ltd., USA, Cat# R2072), after which quantity and quality of the RNA were assessed via a Beckman dual spectrophotometer (USA). A SuperScript IV One-Step RT‒PCR Kit (Thermo Fisher Scientific, MA, USA, Cat# 12594100) was used for reverse transcription of the extracted RNA followed by PCR. A 48-well plate Step One instrument (Applied Biosystems, USA) was used for thermal profiling as follows: 10 min at 45 °C for reverse transcription, 2 min at 98 °C for RT inactivation, and initial denaturation for 40 cycles of 10 s at 98 °C, 10 s at 55 °C, and 30 s at 72 °C for the amplification step. The expression of the sample was normalized to that of the housekeeping gene GAPDH. The TNF-encoding gene sequences of primer sequences were forward 5′- GGAGACCTCGGACCCGTGCA − 3′ and reverse 5′- AAACAACAGATCAAGCACAG − 3′ (gene bank accession number AF329986.1), the sequence of IL- 17 forward 5′- CCGAGATAACTTTGAGGCATA − 3′ and reverse 5′- AACGAGGTTTGACTTTCACA − 3′ (gene bank accession number NM_001106897.1), and the sequence of GAPDH used for the housekeeping gene were forward 5′- CCTCGTCTCATAGACAAGATGGT − 3′ and reverse 5′- GGGTAGAGTCATACTGGAACATG − 3′ (gene bank accession number NM_001394060.2).

### ELISA technique

The intestinal concentrations of Bax (Cat# SEB343Ra, Cloud-clone, TX, USA), Bcl2 (Cat# SEA778Ra, Cloud-clone, TX, USA), caspase- 3 (Cat # E4592 - 100, Biovision, CA, USA), Nrf2 (Cat# RD-NFE2L2-R, Reddot Biotech, TX, USA), and ASK1 (Cat# ER2151, FineTest Biotech Inc., Colardo, USA) were determined using an appropriate ELISA kit according to the manufacturer’s protocols.

### Determination of DNA damage by comet assay

Alkaline single-cell gel electrophoresis was used to measure DNA damage parameters in intestinal tissues using Comet Assay™ Kit (cat. # 4250–050-K, AMS Biotechnology, England, UK) to quantify the number of apoptotic cells. All steps were performed according to the supplier’s instruction (Singh et al. 1988). Images of fluorescence microscopy were captured using a fluorescence microscope (Nikon Eclipse Ti-SR, Tokyo, Japan) that reveals comet-like structures formed by high-pH electrophoresis; the intensity of the comet tail relative to the head represents the amount of DNA breaks. The most likely explanation is that loops with a break lose supercoiling and are free to stretch toward the anode (Ji et al. 2022).

### Statistical analysis

The data are expressed as the mean ± SD. The statistical significance of differences groups was determined by one-way ANOVA followed by Tukey’s multiple comparisons test as a post hoc comparison test. The Kruskal–Wallis test, followed by the post-hoc test (Dunn’s multiple comparisons test) for pairwise comparisons, was used for histopathological evaluations. The acceptable level of significance was *P* < 0.05. All the statistical tests and figures were generated using Graph Pad Prism version 8.0 (Graph Pad Prism Software, CA, USA).

## Results

### Effect of octreotide on histopathological changes in intestinal ischemic reperfused rats

**Fig. 1:**
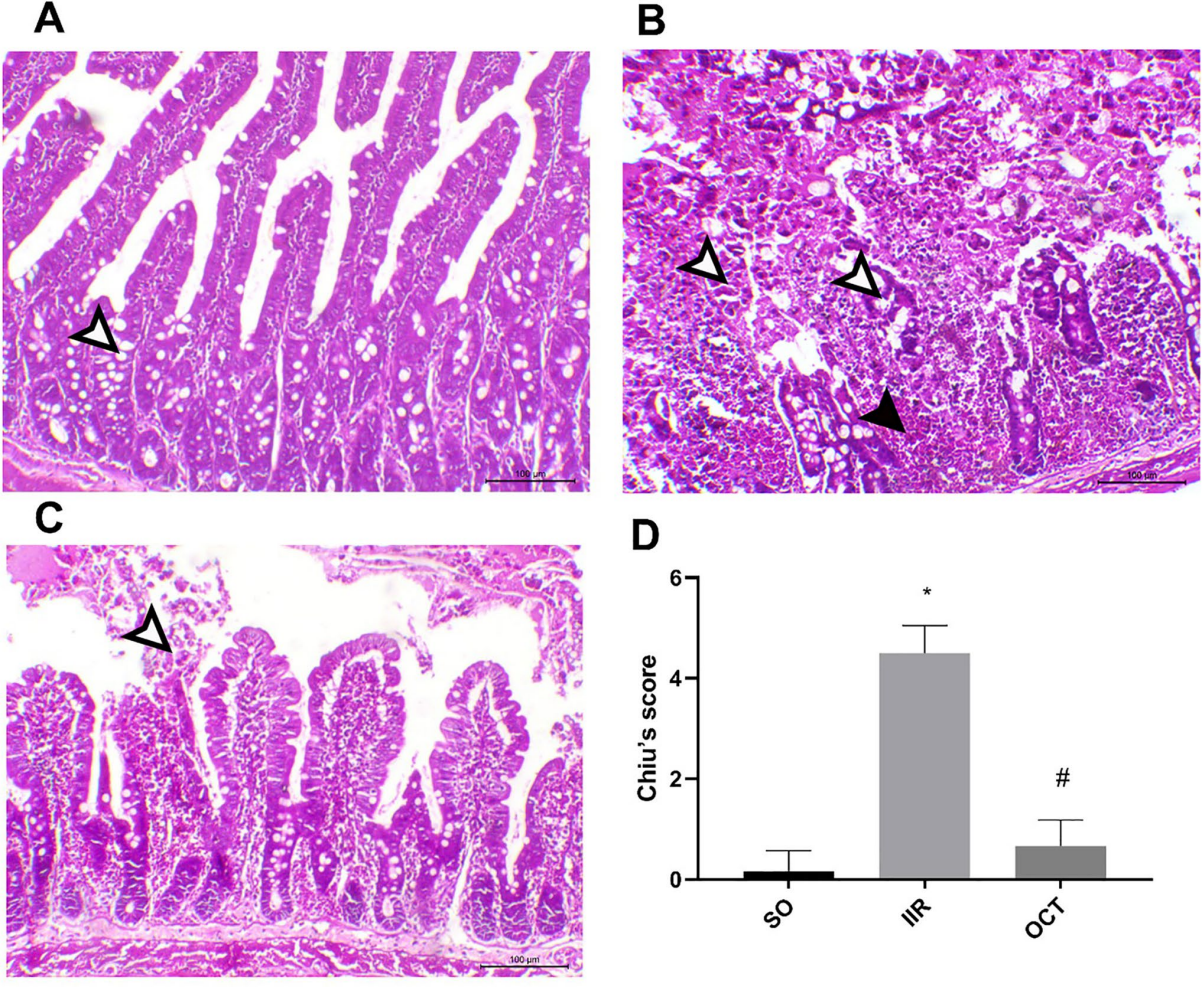
Effect of OCT on histopathological changes in rats subjected to intestinal ischemia/reperfusion injury. The representative photomicrographs of sham rats (**A**) showed normal intestinal villi with numerous goblet cells (white arrowhead). The IIR intestinal sections (**B**) showed severe hemorrhagic necrotic changes along the entire intestinal villi (white arrowhead) mixed with severe hemorrhage within the deep mucosa (black arrowhead). The OCT intestinal sections (**C**) showed mild desquamative changes that were mostly within the apical portion of the intestinal villi (white arrowhead). The panel (**D**) demonstrated the Chiu’s scoring. OCT (50 μg/kg, i.p.) was injected 30 min prior to intestinal IR (60 min/2 h). Results are expressed as mean ± SD (*n* = 6). Statistical analysis was performed using Kruskal–Wallis, followed by Dunn’s post hoc test (*P* < 0.05), as compared with the SO (*) and IIR (#) groups. IIR, intestinal ischemia/reperfusion; OCT, octreotide; SO, sham-operated. Scale Bar = 50 µm for all photomicrographs

As depicted in Fig. 1, panel A shows that the SO rats’ sections had normal mucosal, submucosal, and muscular tissues. The mucosa showed normal intestinal villi that extended deep to form intestinal crypts. The lamina propria showed normal lymphatic and vascular capillaries, and the outer layer showed a normal smooth muscle layer. Panel B shows that the IIR sections had severe hemorrhagic necrotic changes, sloughing, and ulceration of the mucosa. Some intestinal villi showed marked blunting and atrophy, with marked edema in the submucosal connective tissues. The lamina propria showed substantial swelling mixed with neutrophilic inflammatory cell infiltration from bleeding. On the other hand, panel C shows that intestinal sections from OCT group exhibited a marked decrease in necrosis and desquamative changes that were mostly observed within the upper portion of the intestinal villi, while the remaining distal portion of the villi and crypts demonstrated mild catarrhal degenerative changes. Edema and hemorrhage also markedly decreased along the mucosa, lamina propria, and tunica muscularis. The blood vessels were intact, with slight hypertrophy of the endothelial cells.

According to Chiu’s scoring, the IIR group had a significantly greater lesion score (4.5 ± 0.55) than the normal group did. On the other hand, the OCT group exhibited a robust decrease in the intestinal lesion score (0.67 ± 0.52) at *P* < 0.05, as illustrated in panel D.

### Effect of octreotide on the Nrf2/PRX2/ASK1/JNK signaling pathway in intestinal ischemic reperfused rats

**Fig. 2:**
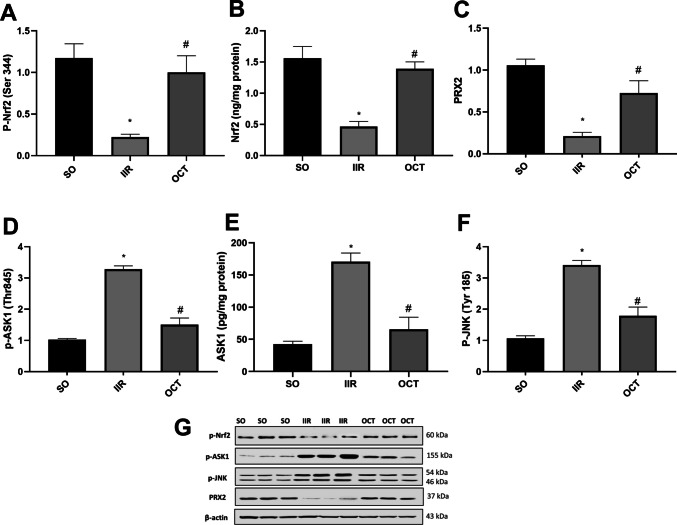
Effect of OCT on **A p-Nrf2**, **B** Nrf2, **C** PRX2, **D p-ASK1**, **E** ASK1, and **F** p-JNK in rats subjected to intestinal ischemia/reperfusion injury. OCT (50 μg/kg, i.p.) was injected 30 min prior to intestinal IR (60 min/2 h). Results are expressed as mean ± SD (*n* = 3). A statistically significant difference at *P* < 0.05 was highlighted with * and # as compared with the SO and IIR groups, respectively. Panel **G** shows the photomicrograph of the western blot assay for the expressions p-Nrf2, p-ASK1, p-JNK, and PRX2, respectively. ASK1, apoptosis signal regulating kinase; JNK, c-Jun N-terminal kinase; IIR, intestinal ischemia/reperfusion; Nrf2, nuclear factor-erythroid 2-related factor 2; OCT, octreotide; PRX2, peroxiredoxin 2; SO, sham-operated

To explore the mechanism underlying the protective effect of OCT, we measured the intestinal protein expression levels of p-Nrf2, PRX2, p-ASK1, and p-JNK using western blot. As illustrated in Fig. 2, compared with SO group, the IIR insult significantly decreased the protein levels of both p-Nrf2 (panels A and G) and its downstream target PRX2 (panels C and G) by approximately 0.2-fold and dramatically increased the levels of p-ASK1 (panels D and G) and p-JNK (panels F and G) by approximately 3.2-fold. However, pre-treatment with OCT significantly increased the levels of p-Nrf2 and PRX2 by 3.5-fold and 2.4-fold, respectively, while simultaneously decreasing the expression of p-ASK1 (46%) and p-JNK (48%) relative to that in the IIR-injured group.

Furthermore, the ELISA results corroborate the western blot findings for ASK1 and Nrf2, where Nrf2 levels (Fig. 2B) decreased from 1.562 ± 0.186 in the SO group to 0.466 ± 0.082 in the IIR group, while ASK1 levels (Fig. 2E) increased from 42.433 ± 4.720 to 170.733 ± 13.332. However, pre-treatment with OCT significantly increased the levels of Nrf2 to 1.390 ± 0.113 and decreased ASK1 to 65.567 ± 18.844, as measured by ELISA. This dual-method approach provides robust evidence for the protective effect of OCT on the Nrf2/PRX2/ASK1/JNK signaling pathway in intestinal ischemic reperfused rats.

### Effect of octreotide on inflammatory markers in intestinal ischemic reperfused rats

**Fig. 3:**
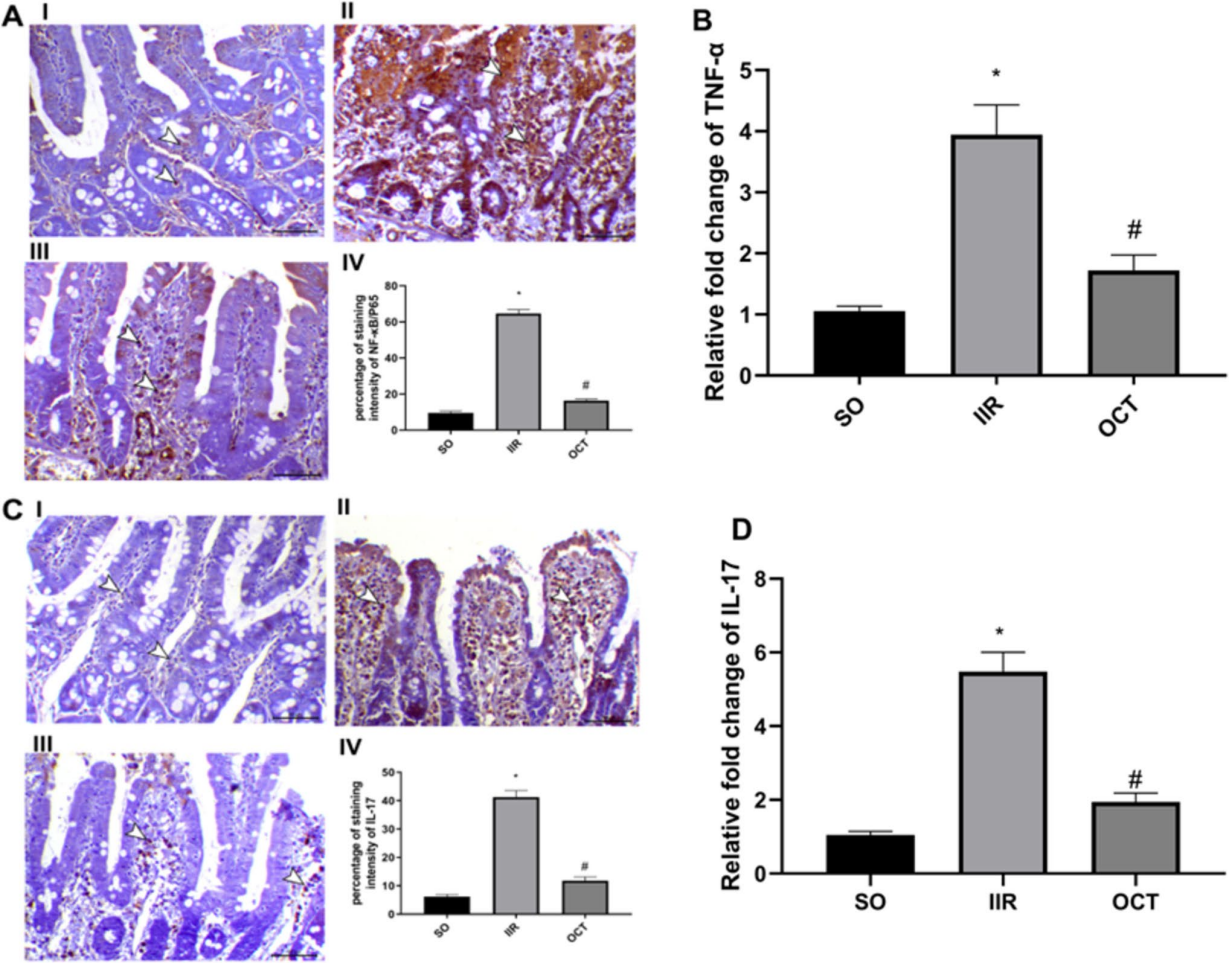
Effect of OCT on **A** NF-кB/P65, **B** TNF-α, and **C**, **D** IL- 17 in rats subjected to intestinal ischemia/reperfusion injury. Panels **A** (I, II, and III) and **C** (I, II, and III) represent the immunohistochemical photomicrographs for NF-кB/P65 and IL- 17, respectively, where I, II, and III represent SO, IIR, and OCT groups, respectively. Panels **B** and **D** represent the gene expressions of TNF-α and IL- 17, respectively. OCT (50 μg/kg, i.p.) was injected 30 min prior to the intestinal IR (60 min/2 h) injury. Results are expressed as mean ± SD (*n* = 6 for PCR analysis and *n* = 4 for immunohistochemical analysis, and arrowheads indicate positive immunostaining). A statistically significant difference at *P* < 0.05 was highlighted with * and # as compared with the SO and IIR groups, respectively. IIR, intestinal ischemia/reperfusion; IL- 17, interleukin- 17; NF-кB, nuclear factor-kappa B 2; OCT, octreotide; SO, sham-operated; TNF-α, tumor necrosis factor alpha. Scale Bar = 50 µm for all photomicrographs

As shown in Fig. 3, the IIR-mediated inflammatory effect augmented the intestinal levels of NF-кB/P65 (panel A-IV), TNF-α (panel B), and IL- 17 (panel C-IV) by 6.8, 3.7, and 6.6-fold, respectively, compared to those in SO group (panels A-I, B, and C-I, respectively). Furthermore, Fig. 3 shows abundant NF-кB/P65 (panel A-II) and IL- 17 (panel C-II) positive cells within the villi and crypt epithelial lining and infiltrated inflammatory cells relative to those in the control group.

However, pre-treatment with OCT significantly reduced the NF-кB/P65 (panel A-IV), TNF-α (panel B), and IL- 17 (panel C-IV) levels by 75%, 56%, and 72%, respectively, when compared to the nontreated IIR group. Additionally, the treated group showed a marked decrease in the immunostaining of NF-кB/P65 (panel A-III) and IL- 17 (panel C-III) within the intestinal mucosa.

The findings of immunohistochemical analysis of IL- 17 were further verified by qPCR, where the SO group showed a baseline IL- 17 mRNA expression level of 1.053 ± 0.090, which increased dramatically to 5.475 ± 0.529 in the IIR group, representing a 5.2-fold increase. OCT pre-treatment significantly reduced IL- 17 mRNA expression to 1.946 ± 0.192, demonstrating a 64.5% reduction compared to the IIR group. Thus, providing testimony for OCT’s anti-inflammatory effects in intestinal ischemia–reperfusion injury at both the protein and mRNA levels, as shown in panel D.

### Effect of octreotide on apoptosis in intestinal ischemic reperfused rats

**Fig. 4:**
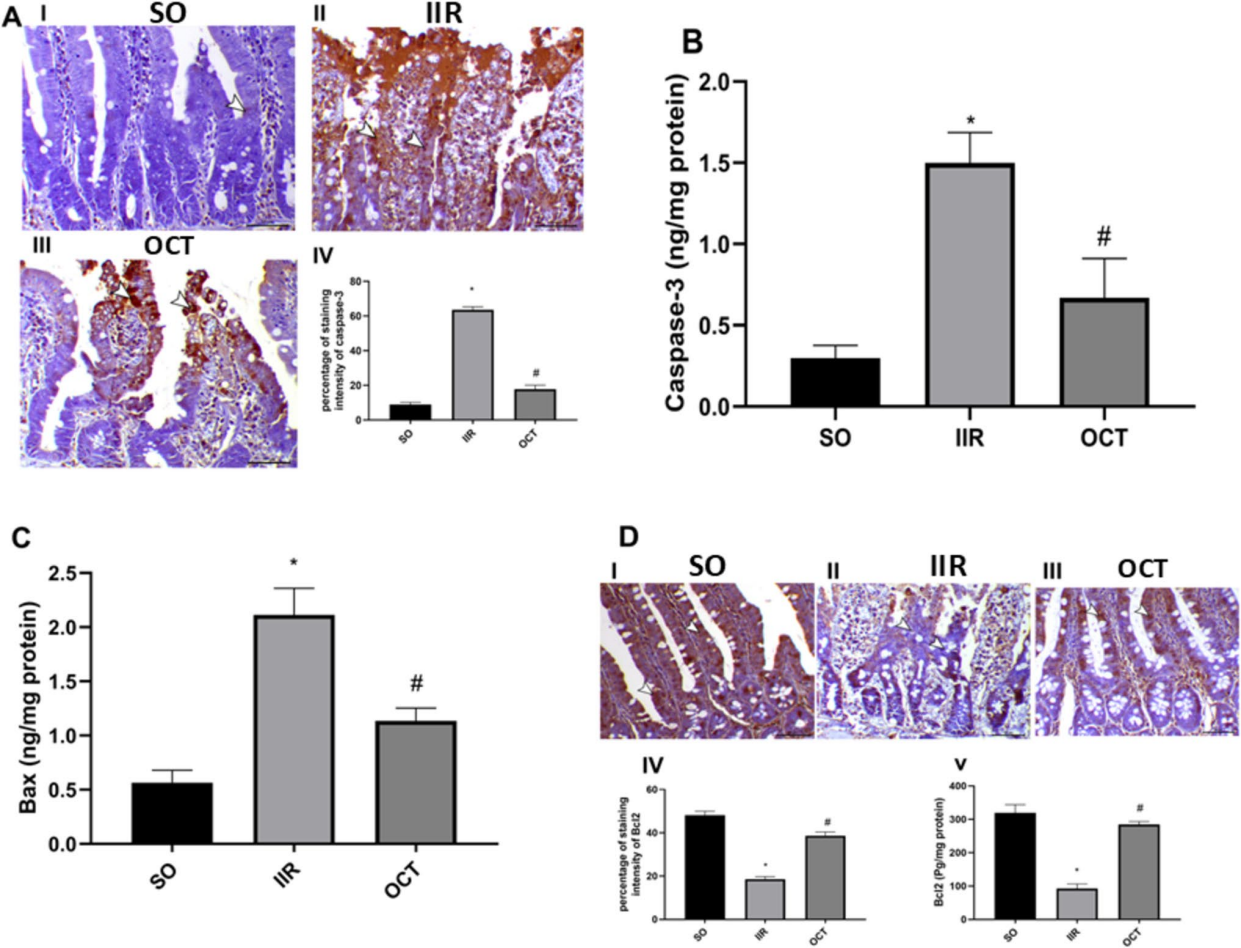
Effect of OCT on **A**, **B** caspase− 3, **C** Bax, and **D** Bcl2 in rats subjected to intestinal ischemia/reperfusion injury. Panels **A** (I, II, and III) and **C** (I, II, and III) represent immunohistochemical photomicrographs for caspase- 3 and Bcl2, respectively. Panels **B**, **C**, and **D**-V represent the protein expression of caspase-3, Bax, and Bcl2 using ELISA, respectively. OCT (50 μg/kg, i.p.) was injected 30 min prior to intestinal IR (60 min/2 h). Results are expressed as mean ± SD (*n* = 6 for ELISA and *n* = 4 for immunohistochemical analysis, and white arrowheads indicate positive immunostaining). A statistically significant difference at *P* < 0.05 was highlighted with * and # as compared with the SO and IIR groups, respectively. Bcl- 2, B cell lymphoma 2; Bax, Bcl- 2-associated X protein; IIR, intestinal ischemia/reperfusion; OCT, octreotide; SO, sham-operated. Scale Bar = 50 µm for all photomicrographs

As shown in Fig. 4A, immunohistochemical staining for caspase- 3 was used to assess apoptosis following IIR injury. The (panel I) SO group showed negative immunoreactivity in the intestinal mucosa (8.9 ± 1.3, *P* < 0.05). In the IIR group (panel II), the dispersed cells had a high degree of immunoreactivity (63.6 ± 1.7, *P* < 0.05). On the contrary, the pre-treatment group (panel III) exhibited a marked decrease in caspase- 3 expression within the intestinal mucosa (17.8 ± 2.3, *P* < 0.05).

These immunohistochemical findings were further confirmed by ELISA results for caspase- 3 (Fig. 4B). The SO group showed a baseline caspase- 3 level of 0.298 ± 0.078, which increased significantly to 1.499 ± 0.187 in the IIR group, representing a 5-fold increase. OCT pre-treatment markedly reduced caspase- 3 levels to 0.670 ± 0.242, demonstrating a 55.3% reduction compared to the IIR group.

Furthermore, Fig. 4 illustrates the apoptotic potential of an IIR insult on the intestine through the increase in the proapoptotic Bax by 3.7-fold (panel C) and the decrease in the anti-apoptotic protein Bcl2 to 29% (panel D-IV and V) compared to the normal baseline. On the other hand, compared with the insult, pre-treatment with OCT corrected the Bcl2/Bax imbalance and inhibited the apoptotic signal by increasing the Bcl2 level by 207% and reducing the Bax level by 46%.

These findings of Bcl2 were consistent with the changes observed via immunohistochemistry (Fig. 4D). The (panel D-II) IIR group showed negative immunoreactivity of Bcl2 within the crypts by 61% when compared to sham. In contrast, the group treated with OCT (panel D-III) exhibited a mitigating response by inducing a 2-fold increase in Bcl2 immuno-expression compared to the injury caused by IIR.

### Effect of octreotide on total antioxidant capacity and superoxide dismutase activity in intestinal ischemic reperfused rats

**Fig. 5:**
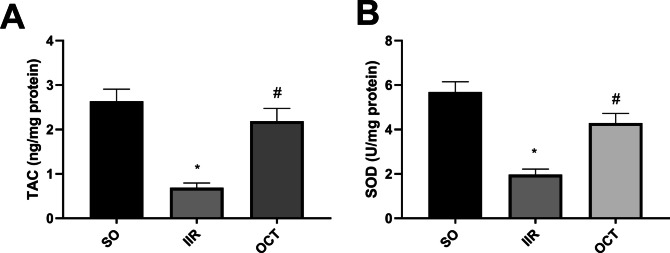
Effect of OCT on **A** TAC and **B** SOD in rats subjected to intestinal ischemia/reperfusion injury. OCT (50 μg/kg, i.p.) was injected 30 min prior to intestinal IR (60 min/2 h). Results are expressed as mean ± SD (*n* = 6). A statistically significant difference at *P* < 0.05 was highlighted with * and # as compared with the SO and IIR groups, respectively. IIR, intestinal ischemia/reperfusion; OCT, octreotide; SO, sham-operated; SOD, superoxide dismutase; TAC, total antioxidant capacity

As shown in Fig. 5, compared with those in the SO group, IIR-associated oxidative stress was confirmed by significant depletion in the intestinal levels of (panel A) total antioxidant capacity (TAC) and (panel B) SOD (74% and 65%, respectively). In contrast, when OCT was administered prior to IIR induction, the TAC and SOD activity dramatically increased by 3-fold and 2-fold, respectively, in comparison with those in the IIR group.

### Effect of octreotide on autophagy in intestinal ischemic reperfused rats

**Fig. 6:**
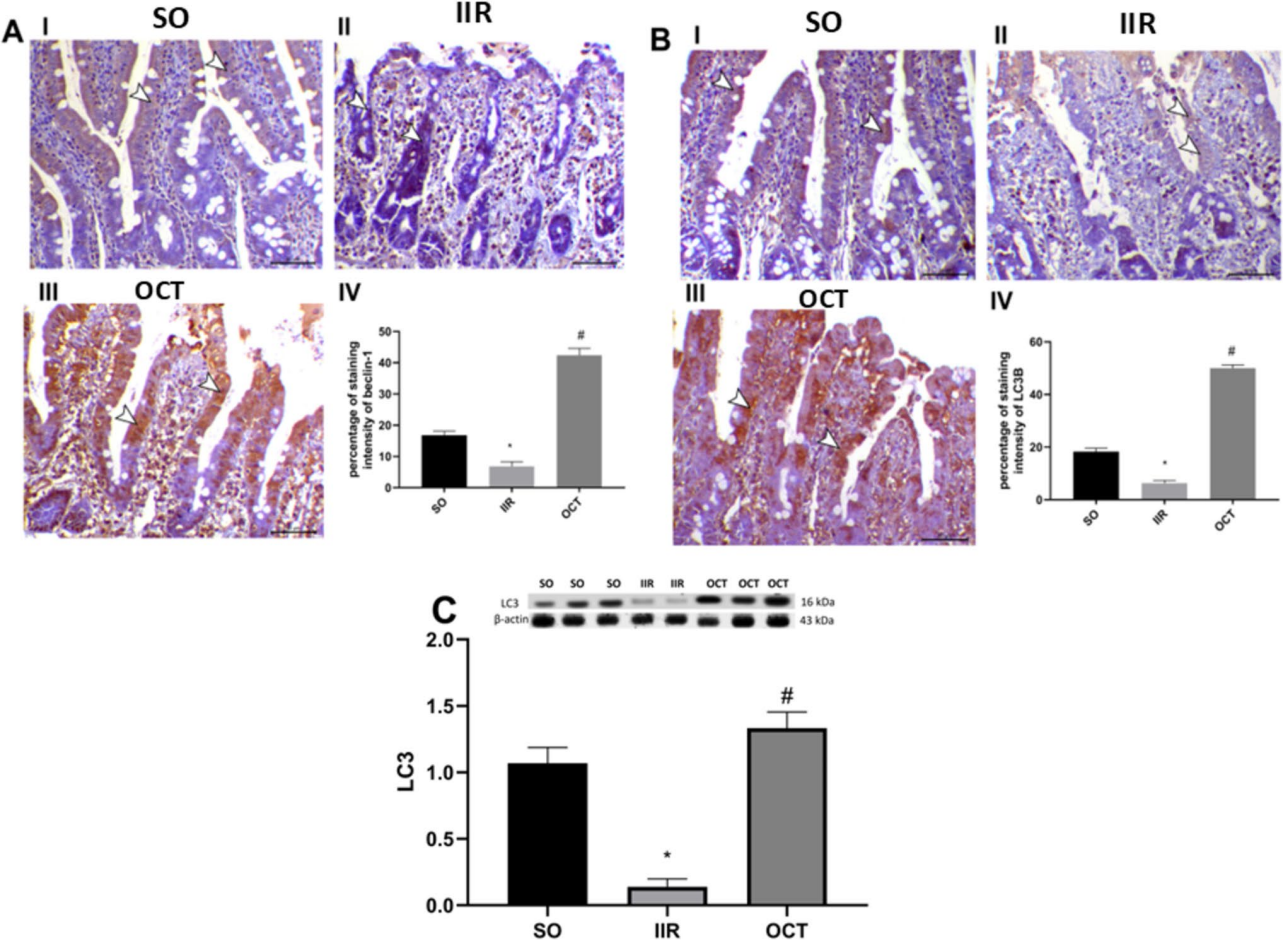
Effect of OCT on **A** beclin- 1 and **B**, **C** LC3 in rats subjected to intestinal ischemia/reperfusion injury. Panels **A** (I, II, and III) and **B **(I, II, and III) represent the immunohistochemical photomicrographs for beclin- 1 and LC3B, respectively (arrowheads indicate positive immunostaining). Panel **C **represents the protein expression of LC3 using western blot technique. OCT (50 μg/kg, i.p.) was injected 30 min prior to intestinal IR (60 min/2 h). Results are expressed as mean ± SD (*n* = 4). A statistically significant difference at *P* < 0.05 was highlighted with * and # as compared with the SO and IIR groups, respectively. IIR, intestinal ischemia/reperfusion; LC3B, light chain; OCT, octreotide; SO, sham-operated. Scale Bar = 50 µm for all photomicrographs

As shown in Fig. 6, IIR insult markedly decreased the immunoreactivity of beclin- 1(A) and LC3B (B) within the necrotic mucosa of both the villi and crypts by 59% and 65%, respectively, indicating the repression of autophagy relative to that in the SO group. Conversely, compared with IIR, OCT significantly increased the autophagic system, as indicated by 6- and 8-fold increases of the immunoexpression of Beclin- 1 and LC3B, respectively.

These immunohistochemical findings of LC3 were further corroborated by western blot analysis. As shown in Fig. 6C, the SO group showed a baseline LC3 level of 1.069 ± 0.118, which decreased significantly to 0.139 ± 0.045 in the IIR group, representing an 87% reduction. OCT treatment markedly increased LC3 levels to 1.333 ± 0.119, demonstrating a 9.6-fold increase compared to the IIR group. These data showed the OCT’s ability to restore autophagy in intestinal ischemia/reperfusion injury.

### Effect of octreotide on intestinal DNA damage in intestinal ischemic reperfused rats

**Fig. 7:**
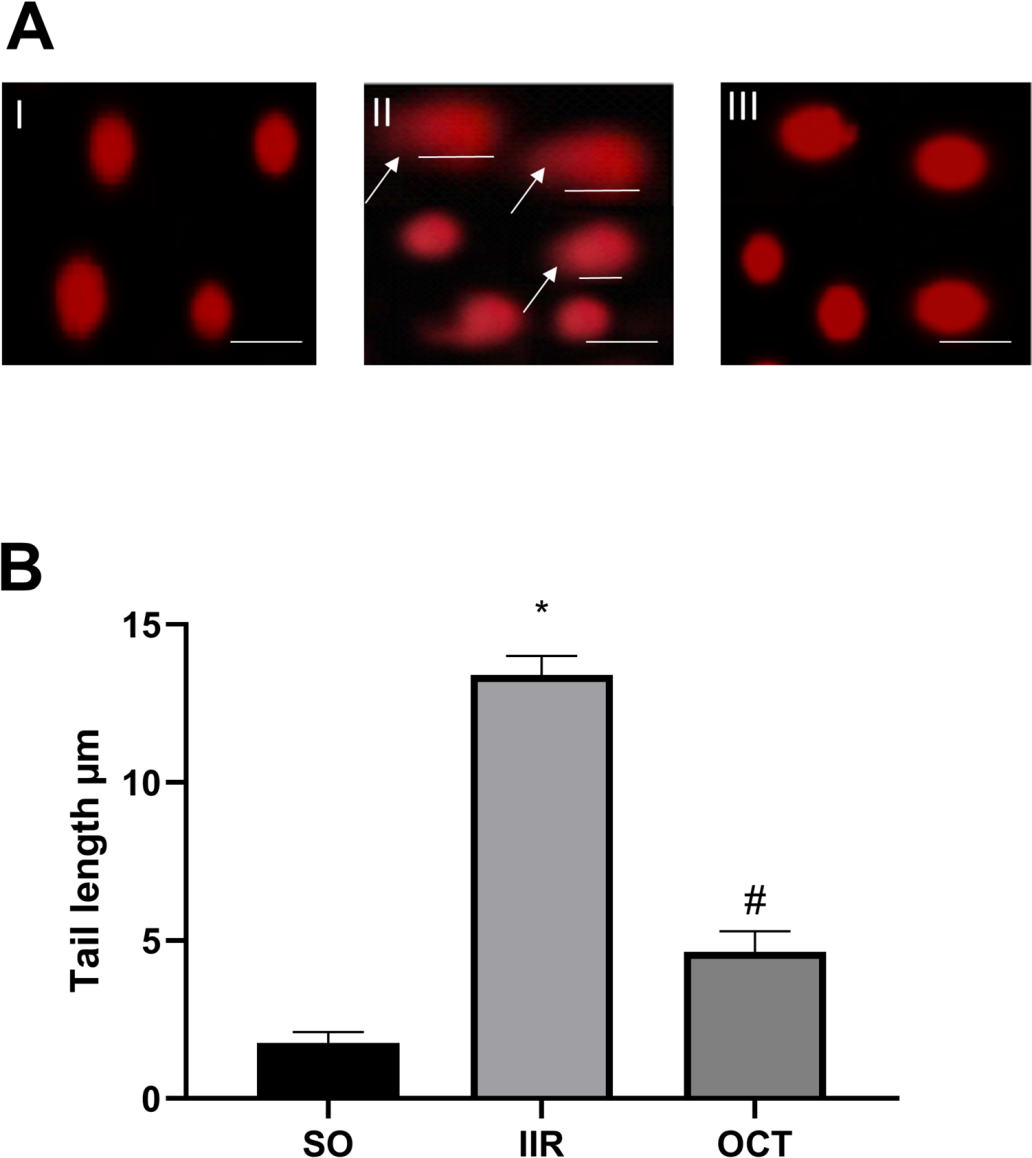
Effect of OCT on intestinal DNA damage in intestinal ischemic reperfused rats. Comet assay showing the degree of DNA damage and tail length in intestinal tissues (**A**-I). Normal control group showing no DNA damage (**A**-II). Intestinal ischemia reperfusion injury group revealing high percent of DNA damage; DNA head at white arrow and DNA tail at white straight line (**A**-III). Octreotide-treated group revealing intact DNA with low DNA damage. Scale Bar = 50 nm. **B** showing histogram statistical analysis of tail length. Results are expressed as mean ± SD (*n* = 3). A statistically significant difference at *P* < 0.05 was highlighted with * and # as compared with the SO and IIR groups, respectively. IIR, intestinal ischemia/reperfusion; OCT, octreotide; SO, sham-operated

The DNA damage in the intestines of rats in different groups was assessed using a comet test. As demonstrated in Fig. 7A and B, compared to SO, the induction group resulted in a substantial increase in DNA damage with a mean value of 18.5% (*p* < 0.05) as evidenced by an increase in tail length to 13.4 µm and percentage of tailed DNA. As the percentage of DNA damage increases, the tail length increases due to the migration of damaged DNA to the tail. In contrast, the administration of OCT antagonized this damage, resulting in lower DNA damage with a mean value of 5.2% and a tail length of 4.65 µm. These results confirm the anti-apoptotic effect of OCT, as reduced DNA damage is closely associated with decreased apoptotic activity in cells.

## Discussion

The current study aims to investigate the precise mechanism underlying the prophylactic effect of OCT against IIR injury. Therefore, it reveals novel findings on the potential signaling crosstalk between the established antioxidant, antiapoptotic, and anti-inflammatory properties of OCT and the newly identified Nrf2/PRX2/ASK1 signaling in alleviating the detrimental effects of IIR insult.

It is well-known that Nrf2-mediated signaling pathway is adopted as one of the crucial signaling pathways that mitigate IIR injury (Piotrowska et al. 2021). It engages with the designated repressor protein Kelch-like ECH-associated protein 1 (Keap1) to establish the Nrf2/Keap1 complex (Yang et al. 2018). In response to oxidative stress, Nrf2 disengages from the Nrf2/Keap1 complex and migrates to the nucleus to maintain the oxidation/antioxidation equilibrium (Tebay et al. 2015; Li et al. 2021).

In the current study, IIR diminished the level of p-Nrf2, which is in line with the findings of an earlier study on IIR injury (Li et al. 2021). However, the OCT group upregulated p-Nrf2 level, which aligns with the findings of prior research in different IR models, including cardiac (Gomaa et al. 2020), brain ischemic stroke (Chen et al. 2012), renal (Mao et al. 2022), and intestinal (Takano et al. 2012) models. Furthermore, Mohamed et al. (2021) revealed the protective effect of OCT against hepatic IR via induction of Nrf2 through the AMPK/PI3K/AKT pathway (Mohamed et al. 2021).

Mounting evidence highlight the protective role of PRX2, a downstream mediator of Nrf2, in eliminating peroxides and inhibiting apoptosis in different disease models, including traumatic brain injury (Zhang et al. 2021), ischemic injury (Leak et al. 2013), Parkinson’s disease (Hu et al. 2011), and subarachnoid hemorrhage (Lu et al. 2019). In this study, IIR successfully suppressed the expression of PRX2. However, the OCT group counterfeited this depletion, which is in agreement with the findings of an earlier study on ischemic neuronal injury (Gan et al. 2012). Intriguingly, no previous studies have investigated the effect of OCT on PRX2, and our study is considered the first to highlight this phenomenon.

The activated ASK1/JNK signaling pathway was confirmed to play a crucial role in worsening IIR injury (Yang et al. 2013). Under stress, ASK1 activates its downstream JNK, causing NF-кB activation (Qiu et al. 2019), consequently increasing inflammatory cytokine release, inducing apoptosis, and ultimately causing cell death (Saitoh et al. 1998; Hayakawa et al. 2012). It was also found that the overexpression of PRX2 inhibits neuronal apoptosis via direct modulation of ASK1 signaling in traumatic brain injury (Zhang et al. 2021). Similarly, our IIR model caused an upsurge of both p-ASK1 and p-JNK levels, compared to the sham group. Nevertheless, OCT administration decreased their levels, and this study was the first to highlight this effect. Interestingly, previous reports elucidated the beneficial role of inhibiting ASK1 in cerebral (Pei et al. 2016) and renal (Sharapov et al. 2020) IR paradigms, which supports our findings. Thus, inhibiting ASK1/JNK pathway may be considered as a promising option for protection against IIR insult.

Mechanistically, activated JNK migrates to the nucleus or mitochondrial membrane, causing the expression of caspase- 3 and other apoptosis-related genes (Dhanasekaran and Reddy 2017), which is recognized as a leading contributor to IIR injury (Giakoustidis et al. 2008; Xiao et al. 2022). Our experimental model revealed a significant increase in JNK and subsequent upregulation of its apoptotic proteins, Bax and caspase- 3, and the inhibition of antiapoptotic marker, Bcl2. Nonetheless, OCT treatment counteracted these effects. Furthermore, the anti-apoptotic effect of OCT was confirmed by the results of comet assay. Similar to our findings, the antiapoptotic attributes of OCT have been demonstrated in hepatic fibrosis induced by carbon tetrachloride (Zhang et al. 2019) and IR injury in either kidney (Xu et al. 2017) or liver (Mohamed et al. 2021). Additionally, several studies confirmed that OCT induces Nrf2 stimulation, which leads to an increase in Bcl2 expression and modulation of the redox state (Chen et al. 2012; Mohamed et al. 2021).

Inflammation and oxidative stress are considered as major contributors to IIR injury (Borges et al. 2016; Jia et al. 2020). NF-кB activation leads to the production of numerous inflammatory agents, such as IL- 17 and TNF-α, and the elevation in ROS levels (Wu et al. 2014; Borges et al. 2016; Zu et al. 2016). Interestingly, NF-кB itself is reactivated by inflammatory mediators and ROS, causing a vicious cycle of inflammation and oxidative stress (Afolabi et al. 2022). Our IIR model showed a significant rise in NF-кB expression, with consequent upregulation of IL- 17 and TNF-α levels, and depletion in TAC and SOD levels, which is in line with an earlier study (Colares et al. 2017).

However, OCT reduced the levels of these inflammatory mediators and caused an increase in TAC and SOD levels as consistent with the findings of previous investigations (Chen et al. 2012; Mohamed et al. 2021). Furthermore, the anti-inflammatory and antioxidant effects of OCT were verified in different IR models, including those of the heart (Khalifa et al. 2022), liver (Mohamed et al. 2021), and kidney (Saleem et al. 2020). Interestingly, a decrease in ROS generation coincides with Nrf2 axis stimulation and NF-кB hub inhibition in intestinal tissue (Reziwan et al. 2019; Jia et al. 2020). Thus, the ability of OCT to prevent apoptosis and reduce inflammation is associated with its impact on Nrf2 activation, and NF-кB and ASK1 inhibition.

Autophagy is a ubiquitous biological process in which fragmented or unnecessary proteins are engulfed and digested in autophagic vesicles (Kimura et al. 2017; Gustafsson and Dorn 2019). The significance of autophagy in the mechanism of IR has been the subject of debate (Wen et al. 2019; Chen et al. 2020; Zhenzhen et al. 2022). While some researchers believe that autophagy induction is detrimental to IIR (El-Malkey et al. 2021; Zhenzhen et al. 2022), others believe that it provides protection against IIR (Wang et al. 2019; Wen et al. 2019). Our study supports the latter point of view, where autophagy markers, LC3B and beclin- 1, were inhibited by IIR, leading to detrimental effects of IIR injury, which is concurrent with the findings of other studies (Wen et al. 2019; Chen et al. 2020; Li et al. 2022). On the contrary, OCT pre-treatment significantly induced autophagy, as indicated by the upregulation of LC3B and beclin- 1 levels, which is inconsistent with other IR models (Sun et al. 2017; Zou et al. 2018). Notably, the activation of Nrf2 signal pathway plays a role in induction of autophagy (Liu et al. 2023). Similarly, the octreotide ameliorates liver IR injury through Nrf2 activation-mediated induction of autophagy (Mohamed et al. 2021).These observations indicate that autophagy could exert a noteworthy influence on the safeguarding effects of OCT against IIR-induced damage.

There are several limitations to consider in our study. Further experimental studies are needed to explore the impact of varying doses of OCT, as they may yield diverse effects. Furthermore, future research is needed to explore OCT’s potential as a post-IIR therapeutic agent. Additionally, our research was founded on an animal model; however, there is a need for clinical applications.

## Conclusion

**Fig. 8:**
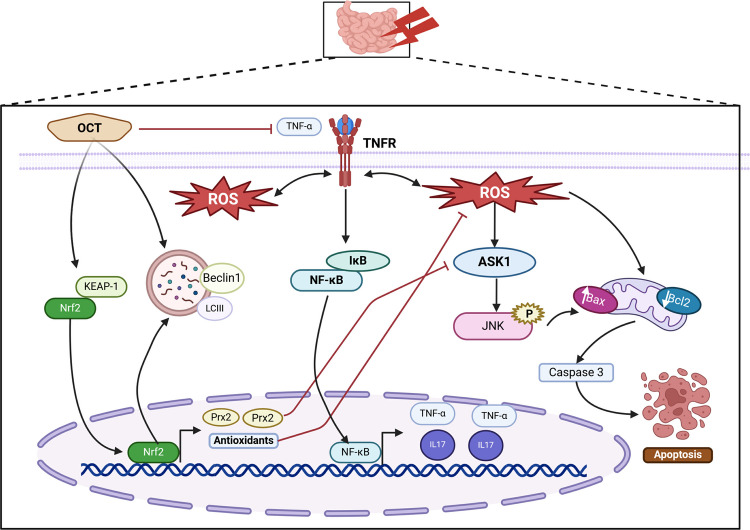
The proposed mechanism of OCT against intestinal ischemia/reperfusion injury. ASK1, apoptosis signal regulating kinase; Bcl- 2: B cell lymphoma 2; Bax: Bcl- 2-associated X protein; IIR, intestinal ischemia/reperfusion; IL- 17, interleunin- 17; JNK, c-Jun N-terminal kinase; Keap 1, Kelch-like ECH-associated protein 1; LC3, light chain; Nrf2, nuclear factor-erythroid 2-related factor 2; NF-кB, nuclear factor-kappa B; ROS, reactive oxidative stress; TNF-α, tumor necrosis factor alpha; TNFR, tumor necrosis factor receptor

The present study highlights the possible novel effect of OCT on the Nrf2/PRX2/ASK1/JNK pathway through its anti-inflammatory, anti-apoptotic, and antioxidant properties, as well as its autophagic role in protecting the IIR insult as illustrated in Fig. 8.

## Supplementary Information

Below is the link to the electronic supplementary material.

Supplementary file1 (PDF 2258 KB)

## Data Availability

All source data for this work (or generated in this study) are available upon reasonable request.
