# Supplementary figures and images for: Octreotide attenuates intestinal ischemia/reperfusion mischief in rats through modulation of Nrf2/PRX2/ASK1/JNK signaling pathway
Source: Naunyn Schmiedebergs Arch Pharmacol. 2025 May 1;398(11):15307–20. doi: 10.1007/s00210-025-04157-0 (PMC12552316; doi:10.1007/s00210-025-04157-0)

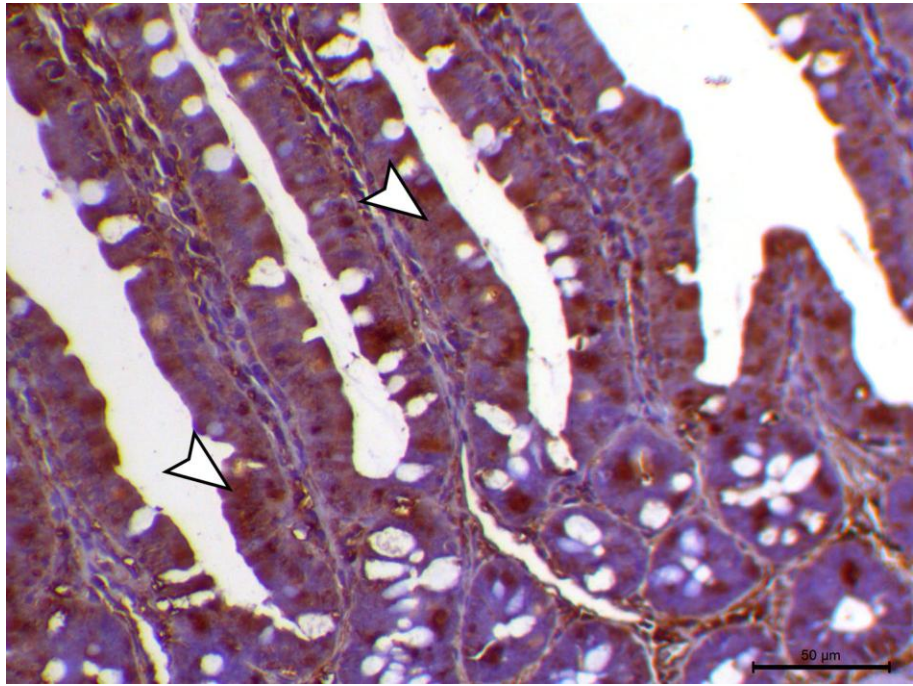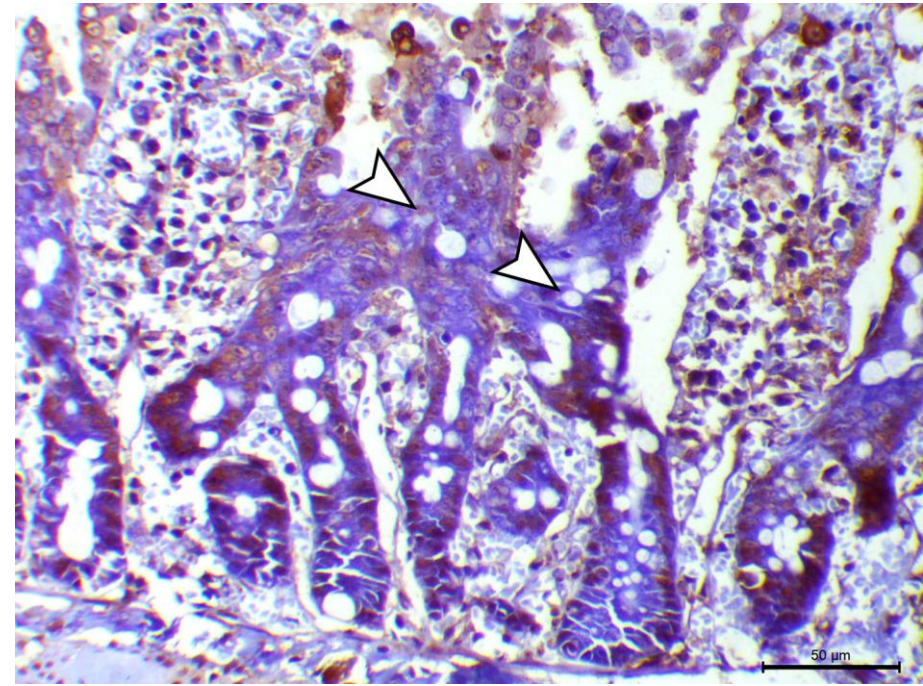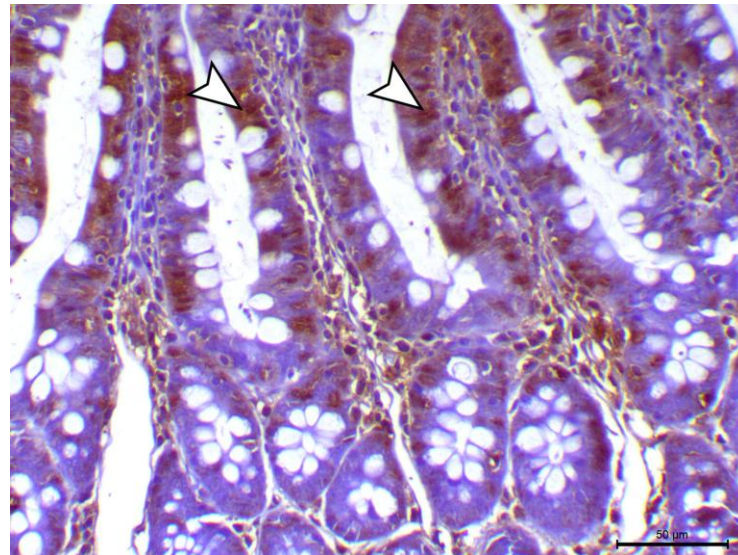

BCL2

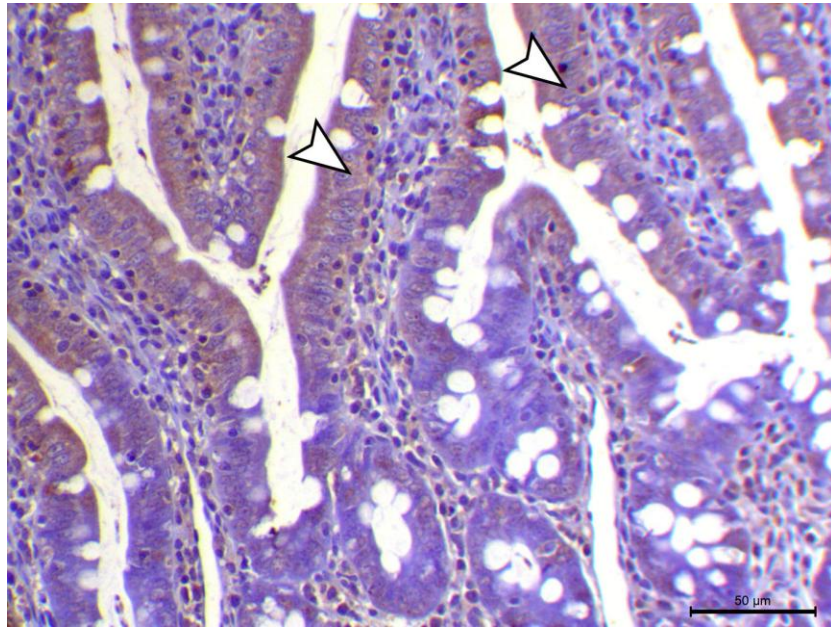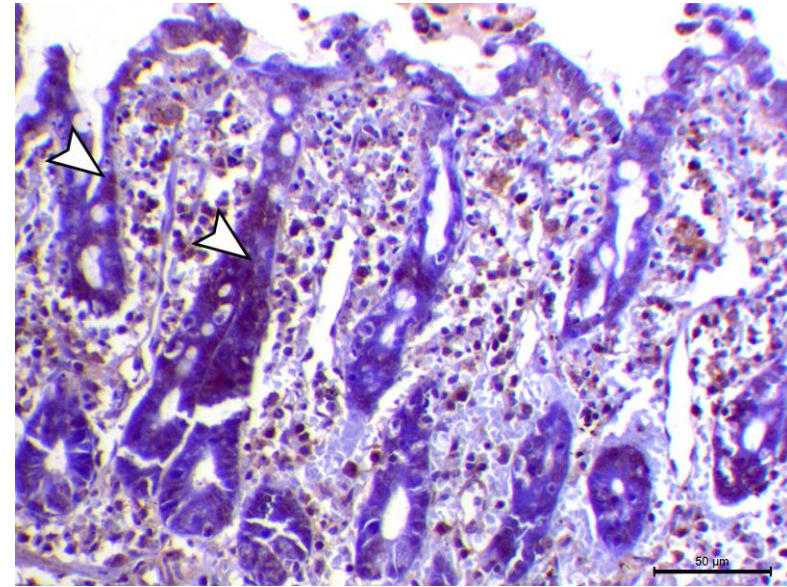

beclin

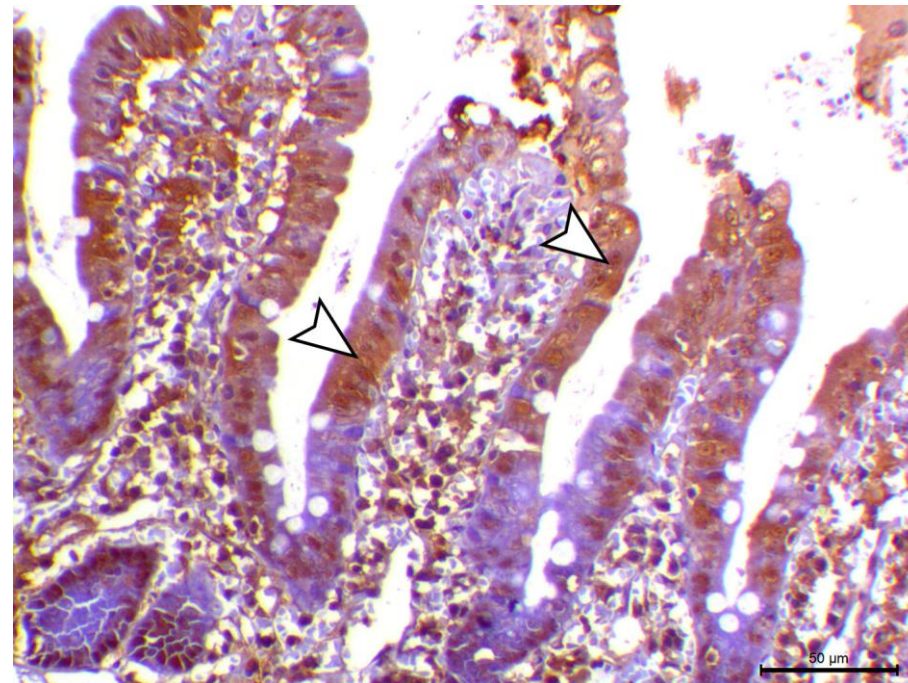

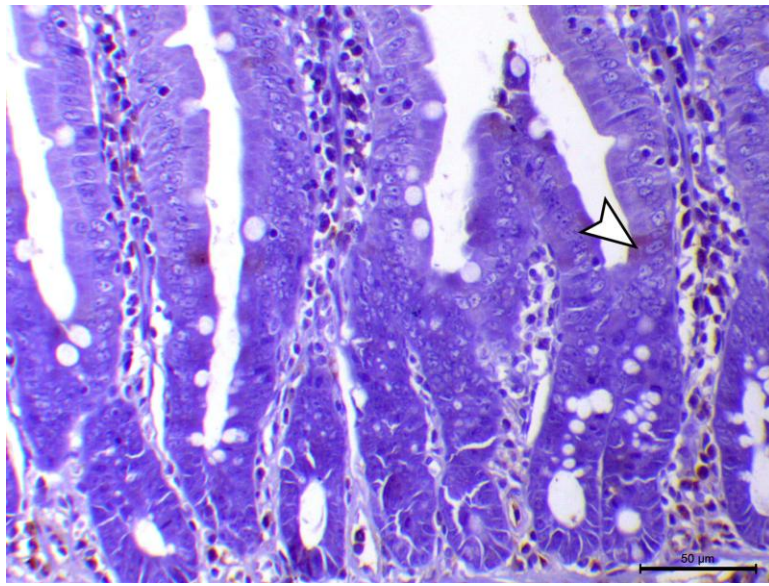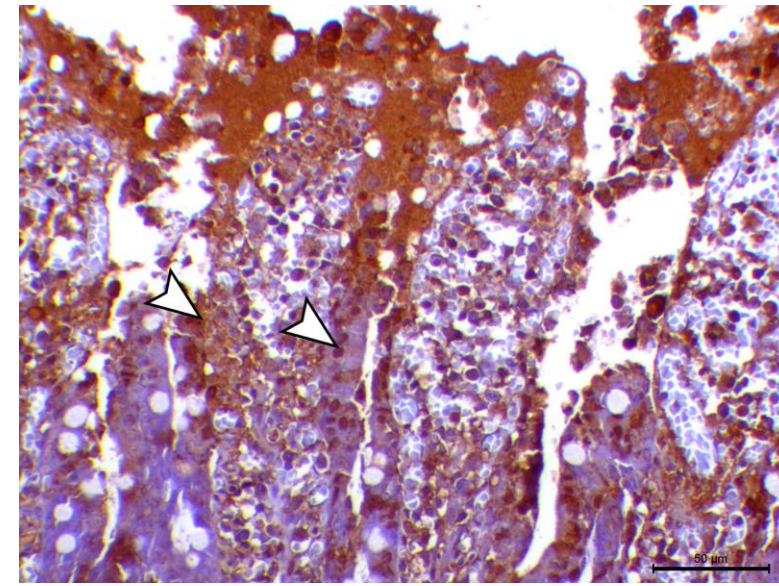

Caspase 3

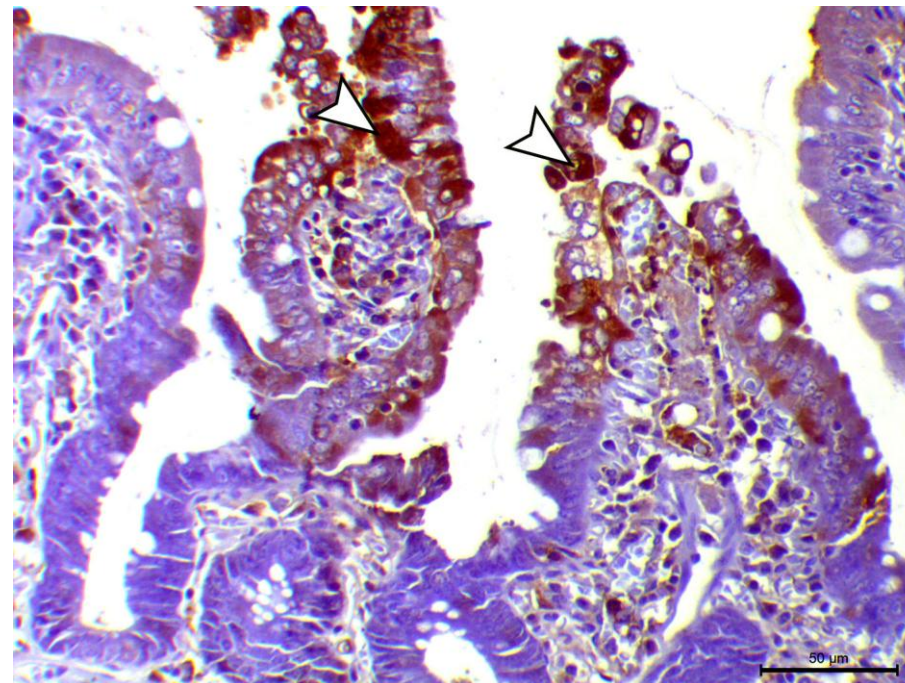

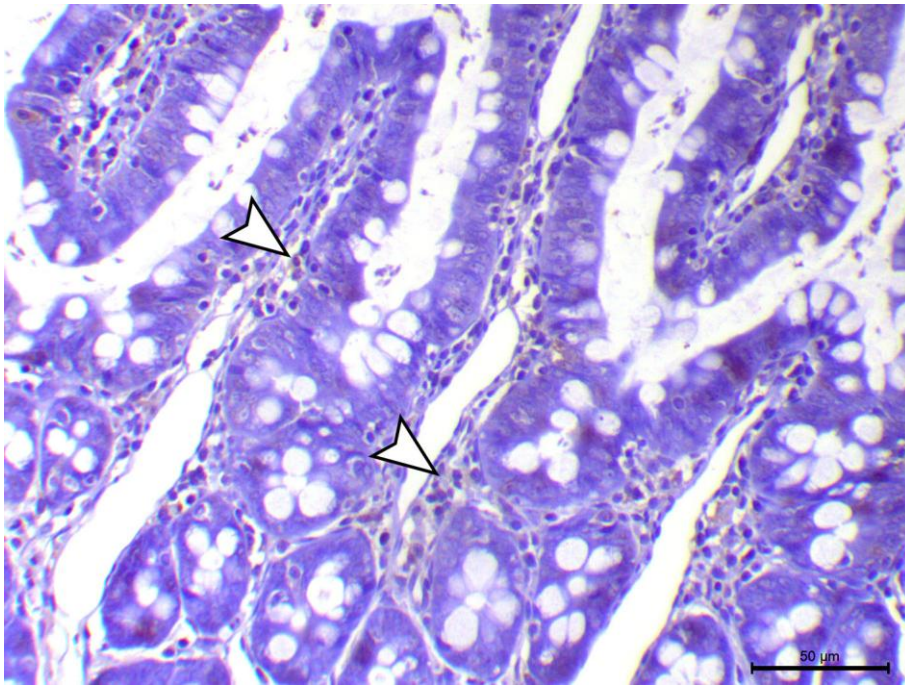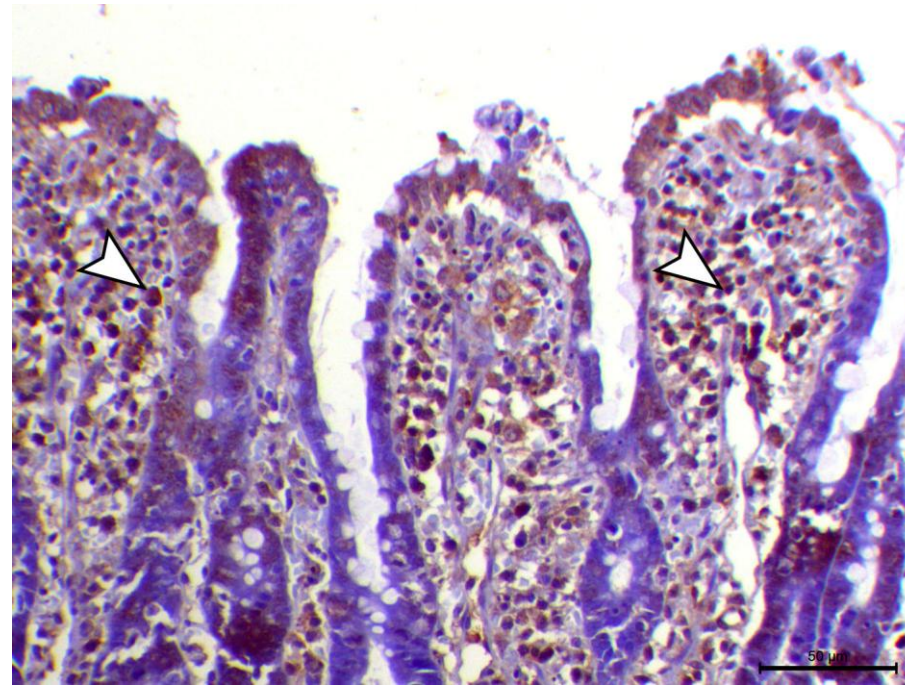

IL-17

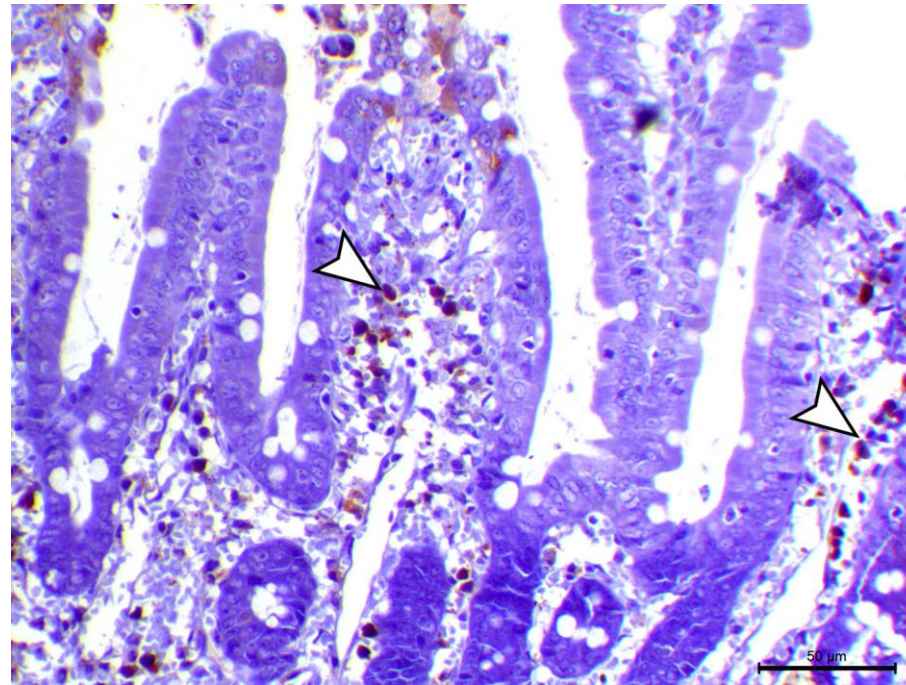

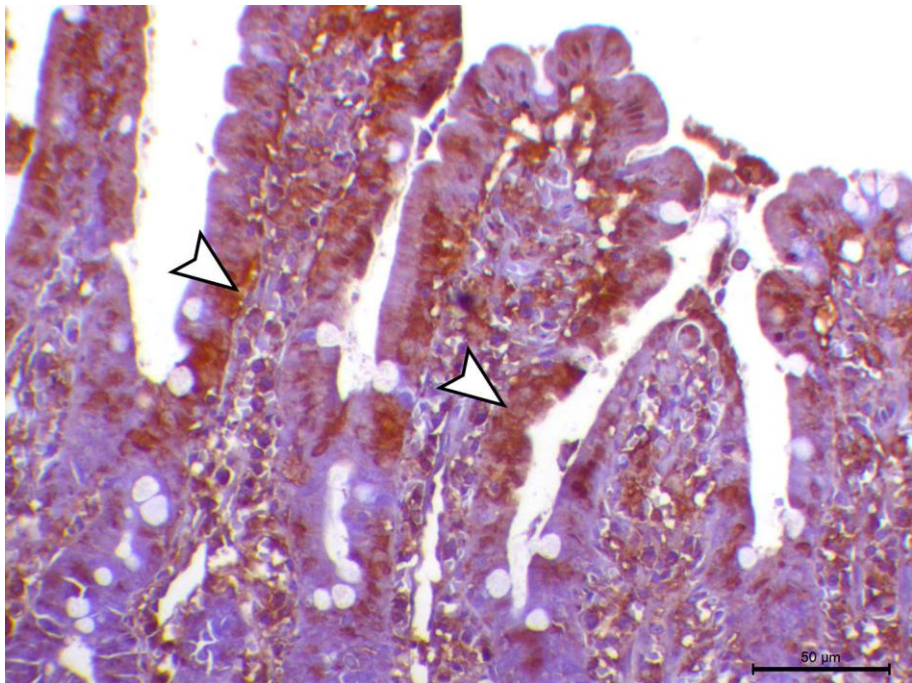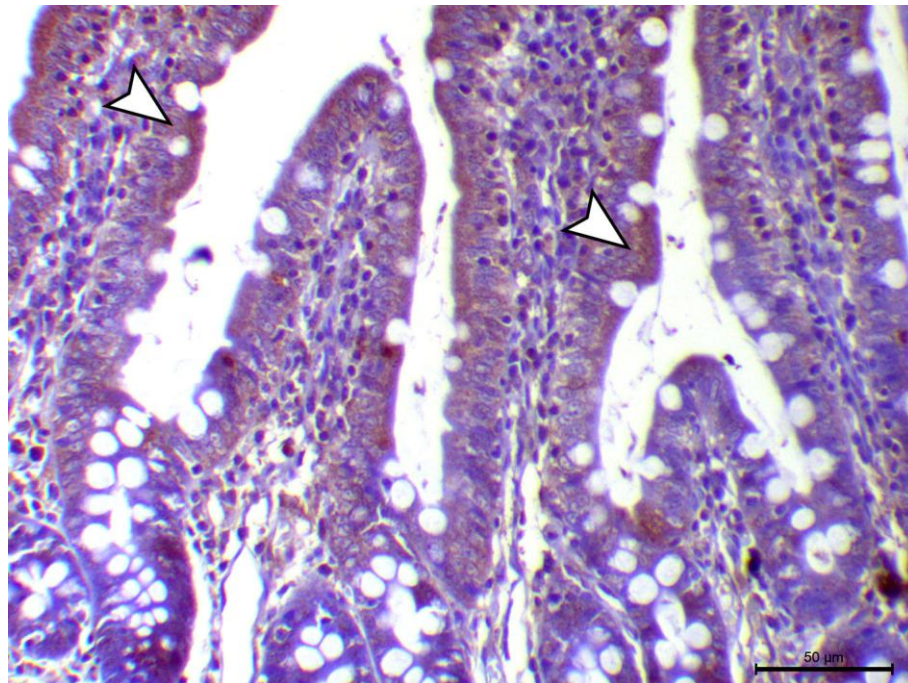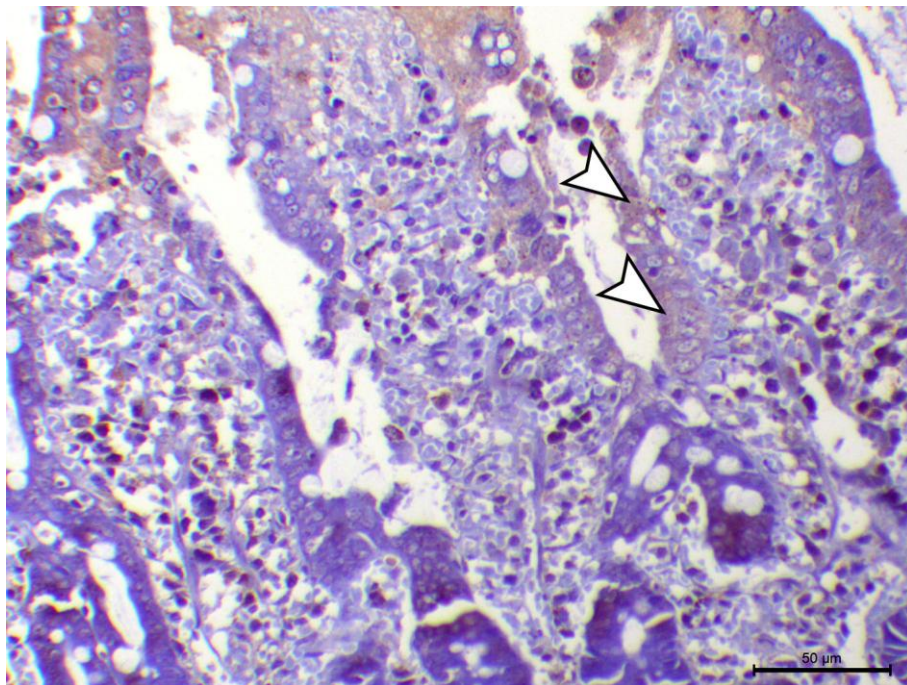

LC3

NF- $\kappa$ B

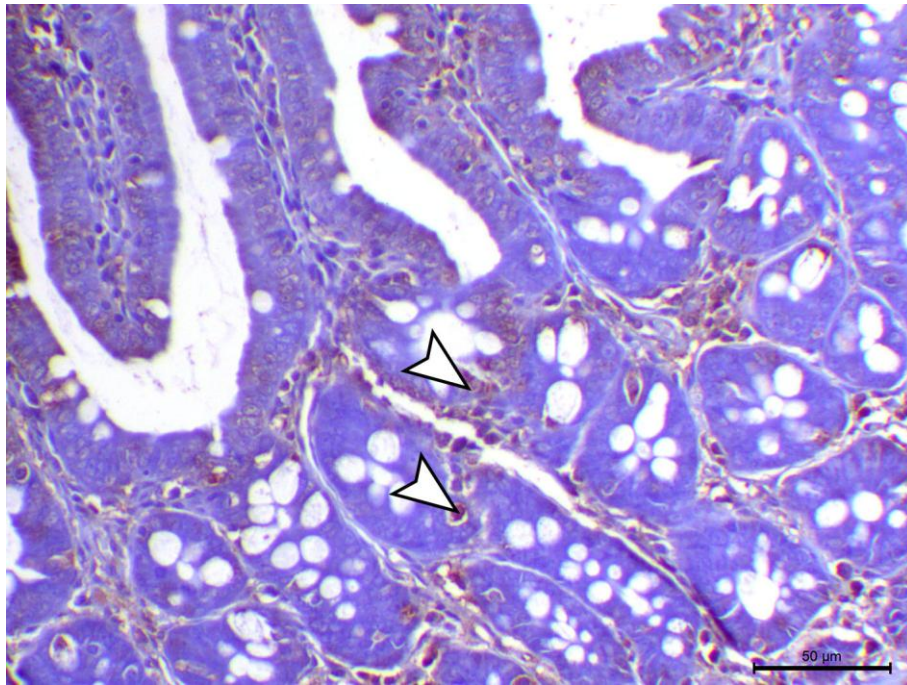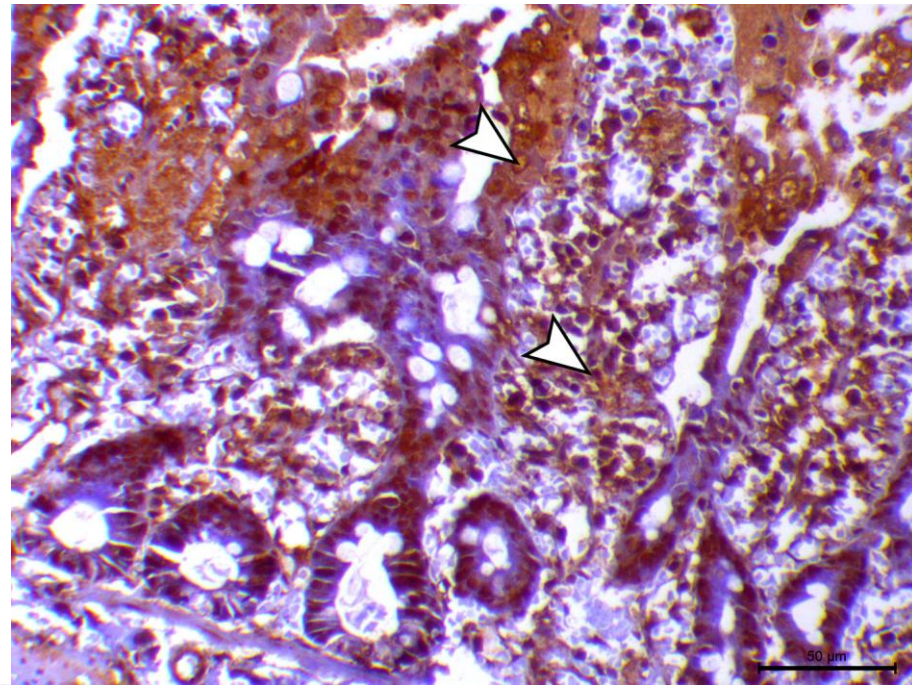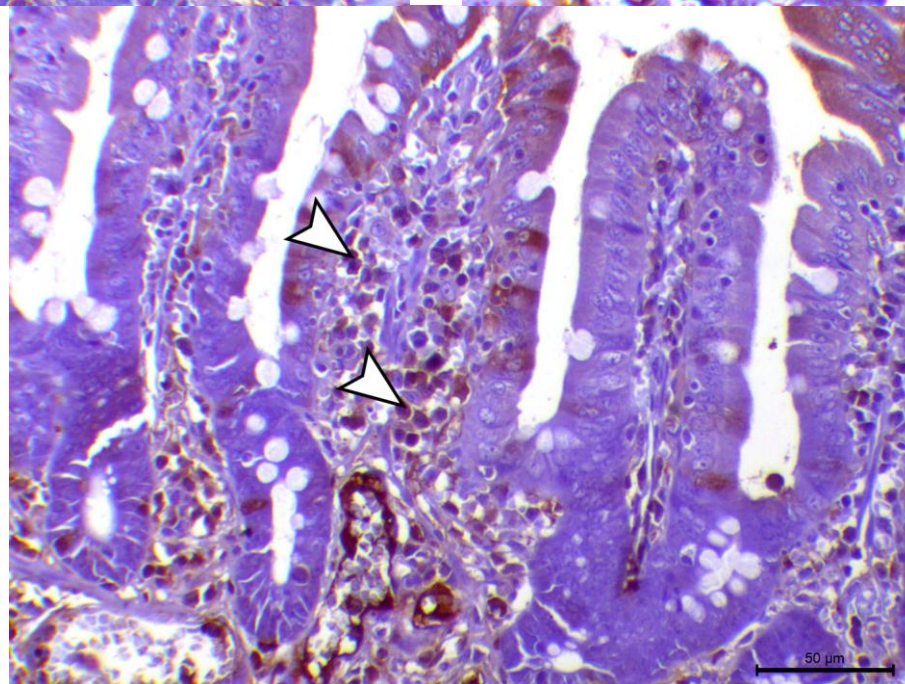

Supplement: Supplementary file 1 — Supplementary file1 (PDF 2258 KB) [file 210_2025_4157_MOESM1_ESM.pdf]
